# Biomarkers in Alzheimer's Disease and Lewy Body Disorders with Dementia

**DOI:** 10.1155/2013/473181

**Published:** 2013-01-09

**Authors:** Thomas Leyhe, Taher Darreh-Shori, Christoph Laske, Michelle M. Mielke, Walter Maetzler

**Affiliations:** ^1^Department of Psychiatry and Psychotherapy, University Hospital of Tuebingen, Calwer Str. 14, 72076 Tuebingen, Germany; ^2^Alzheimer Neurobiology Center, Department of Neurobiology, Care Sciences and Society, Karolinska Institutet, 141 86 Stockholm, Sweden; ^3^German Center for Neurodegenerative Diseases (DZNE), Otfried Müller-Strasse 27, 72076 Tuebingen, Germany; ^4^Section for Dementia Research, Hertie Institute of Clinical Brain Research and Department of Psychiatry and Psychotherapy, University of Tuebingen, Calwer Str. 14, 72076 Tuebingen, Germany; ^5^Department of Health Sciences Research, Mayo Clinic, 200 First Street SW, Rochester, MN 55905, USA; ^6^Department of Neurodegeneration, Center of Neurology and Hertie Institute for Clinical Brain Research, Hoppe Seyler-Straße 3, University of Tuebingen, 72076 Tuebingen, Germany

The present special issue provides a synthesis of current materials and techniques used to not only explore, but also to validate and evaluate novel markers, potentially new biomarkers, in the field of dementias associated with Alzheimer's and Lewy body pathology. This topic is of particular interest as only a very minor proportion of potential markers detected in preclinical studies will ultimately be included in larger clinical studies or for routine clinical use. 

The review article by Wang et al. focuses on an exciting and novel topic in the field, the potential of the quantification of small brain protein degradation fragments in blood, which are generated specifically by brain-derived proteases, to diagnose and follow neurodegenerative processes. The authors thoroughly review the currently available literature. They then speculate on the utility of this strategy as a method of choice for the development of novel biomarkers for disease progression, stage of disease, and treatment efficacy in large cohorts of demented patients as well as individuals at risk for neurodegenerative dementias. A specific focus is on the potential of tau-, APP-, and alpha-synuclein-associated fragments.

The review of Deleidi and Maetzler argues that disturbances in protein clearance mechanisms in the brain substantially contribute to neurodegenerative dementias associated with Abeta and Lewy body pathology. In fact, much evidence has been accumulated pointing to defective protein clearance mechanisms involved in the initiation and progress of sporadic Alzheimer's disease. The authors provide, for the first time, an extensive overview of the literature dealing with protein clearance aspects in the field of Lewy body-associated dementias, with a particular focus on intraneuronal and extraneuronal clearance mechanisms of Abeta and alpha-synuclein.

Alpha-synuclein, and the detection of its monomeric and oligomeric species in cerebrospinal fluid and blood of patients with dementia with Lewy bodies, is also the central focus of the review provided by Kasuga et al. The authors conclude that the determination of altered alpha-synuclein species such as truncated, phosphorylated, and oligomeric forms in body fluids may have a far higher potential to differentiate between healthy and disease state compared to the measurement of unaltered alpha-synuclein levels.

In addition to the review papers, there are several research papers included in this special issue highlighting new research in the field. Baldin et al. assess the associations between neuropsychological outcomes and pro- and anti-inflammatory serum markers in a large cohort of patients with Alzheimer's disease and controls. Their results suggest that individuals with cognitive impairment possess different proinflammatory and anti-inflammatory “strategies” compared to those without. The authors argue that combinations of inflammation and neuropsychological measures, as presented in their study, may improve diagnostic quality. As this is a baseline assessment of a longitudinal study, we expect that the initial results concerning the predictive diagnostic value of this approach—which are even more interesting—will soon be available. This article fits well in a rapidly growing number of important articles reporting about the usefulness of *combinations* of outcome measures to define state, trait, and risk of chronic (neurodegenerative) diseases.

Schreitmüller et al. assess vascular markers by Alzheimer's disease severity. Their research is based on the idea that in Alzheimer's disease a continuous vascular activation, induced by hypoperfusion and factors and processes associated with angiogenesis, occurs. In their study, the authors investigated serum levels of the proangiogenic factor Angiopoietin-1 in patients with Alzheimer's disease, mild cognitive impairment, and controls. They found higher levels in patients with Alzheimer's disease and concluded that Angiopoietin-1 is a potential candidate for a respective biomarker panel.

Duchesne et al. provide another (if not the most important) piece in the biomarkers' development puzzle. They nicely illustrate the importance of implementing adequate verification, validation, and evaluation steps before a promising marker can be integrated into large scale studies or even routine clinical assessment. More specifically, they report both the within-session scan/repeat and across-session scan/rescan reliability (a component of validity) of a self-developed assessment tool that detects medial temporal lobe atrophy via MRI images. In their article, they end up with a power calculation, that is, an estimation of minimum precision threshold that must be added to the effect size, to obtain true cohort sizes for clinical trials.

Lastly, Liepelt-Scarfone et al. report on the diagnostic utility of neuropsychological test results obtained from a large cohort of nondemented and demented patients with Parkinson's disease. In this paper, the authors introduce factor and cluster analysis strategies that enable a differentiation between different subtypes of Parkinson's disease patients, a detection of similarities between test results, and a reduction of a large number of markers on an explorative, hypothesis-free basis. These are particularly important approaches to increase the quality of clinical data that are included in biomarker studies, and to improve our understanding about pheno- and endophenotypes in respective disorders.

It must be emphasized that the evaluation of any therapy is heavily depending on the existence of sufficient trait and state markers which are currently, in particular in Lewy body disorders, not available. With this respect, we feel that this special issue offers both review articles and original research which provide a comprehensive overview of promising (combinations of) biomarkers as well as state-of-the-art techniques to evaluate them. We hope this issue will stimulate the continuing efforts towards developing promising and reliable markers for these particularly burdensome disorders.

*Thomas Leyhe* *Thomas Leyhe* *Taher Darreh-Shori* *Taher Darreh-Shori* *Christoph Laske* *Christoph Laske* *Michelle M. Mielke* *Michelle M. Mielke* *Walter Maetzler* *Walter Maetzler*

